# Exploratory analysis of associations between postpartum body condition changes measured by an automated 3-dimensional camera and reproductive outcomes measured by in-line milk progesterone analysis

**DOI:** 10.3168/jdsc.2025-0773

**Published:** 2026-01-16

**Authors:** Alessandro Frizza, Erminio Trevisi, Luca Cattaneo

**Affiliations:** 1 Department of Animal Science, Food and Nutrition (DIANA), Faculty of Agricultural, Food and Environmental Sciences, Università Cattolica del Sacro Cuore, 29122 Piacenza, Italy; 2 Romeo and Enrica Invernizzi Research Center for Sustainable Dairy Production of the Università Cattolica del Sacro Cuore (CREI), 29122 Piacenza, Italy

## Abstract

Our objective was to evaluate the associations of postpartum BCS changes, as predicted by automatic monitoring using 3-dimensional (3D) camera technology after calving, with milk yield and reproductive outcomes assessed both from in-line milk progesterone analysis and conventional methods in Holstein dairy cows in a retrospective observational study. Cows calving in a commercial farm during a whole year (n = 123; 53 primiparous and 70 multiparous) were automatically evaluated for BCS with a 3D camera (DeLaval Body Condition Scoring system) daily on a 5-point scale up to 120 DIM and retrospectively classified into tertiles based on BCS change from calving to 30 DIM. The resulting groups had high (HI), intermediate (IN), and low (LO) BCS change. Milk yield and milking frequency were collected daily from the automatic milking system (VMS V310, DeLaval), which also automatically evaluated milk progesterone concentration every 2 to 3 d on average. These data were used to characterize luteal activity, with progesterone concentrations peaking during luteal phases and reaching a nadir around estrus events, thus allowing identification of the resumption of cyclicity. Pregnancy outcomes were evaluated up to 200 DIM with logistic regressions and time-to-event data with Cox's proportional hazard models. Continuous data were analyzed with ANOVA, with repeated measures mixed models when appropriate. The models included the effects of BCS tertile and parity. Body condition score loss averaged −0.47, −0.30, and −0.09 for HI, IN, and LO, respectively, ranging from −0.78 to −0.36 in HI, from −0.35 to −0.25 in IN, and from −0.24 to 0.41 in LO. Cows with minimal BCS loss (LO) were thinner (BCS = 3.23 ± 0.29) at calving than cows with greater BCS loss (HI and IN, BCS = 3.38 ± 0.25 and 3.37 ± 0.23, respectively). The BCS nadir was lower in HI compared with IN and LO, although the time to the nadir did not differ. The HI cows tended to have later commencement of luteal activity (i.e., DIM of the first of at least 2 consecutive samples with milk progesterone ≥5 ng/mL; +8 d vs. IN and LO), calculated based on progesterone profiles, but a similar percentage of cows (∼95% overall) resumed ovarian cyclicity before the end of voluntary waiting period (75 DIM). The proportion of cows pregnant at the first artificial insemination (AI) did not differ, but LO cows tended to have increased likelihood of pregnancy at the second AI compared with IN. The cumulative proportion of pregnant cows at the first and second AI tended to be greater in LO compared with HI and IN, and the proportion of pregnant cows up to 200 DIM was greater in LO. Compared with LO, the HI and IN cows had reduced hazard of pregnancy up to 200 DIM. Milk yield was 4 and 3 kg/d lower in LO compared with HI and IN. Automated BCS and progesterone monitoring showed that greater BCS loss in the first 28 DIM was associated with delayed cyclicity and reduced reproductive performance, whereas minimal loss was associated with greater reproductive success but lower yield.

## Highlights

• Postpartum BCS change was evaluated with an automated 3-dimensional camera.
• In-line milk progesterone profiles were used to characterize luteal activity.
• Postpartum BCS loss was associated with delayed cyclicity and fertility loss.
• Cows with minimal BCS loss had improved fertility but lower milk yield.
• Automated phenotyping shows potential for precision dairy herd management.

## Graphical Abstract

**Figure:**
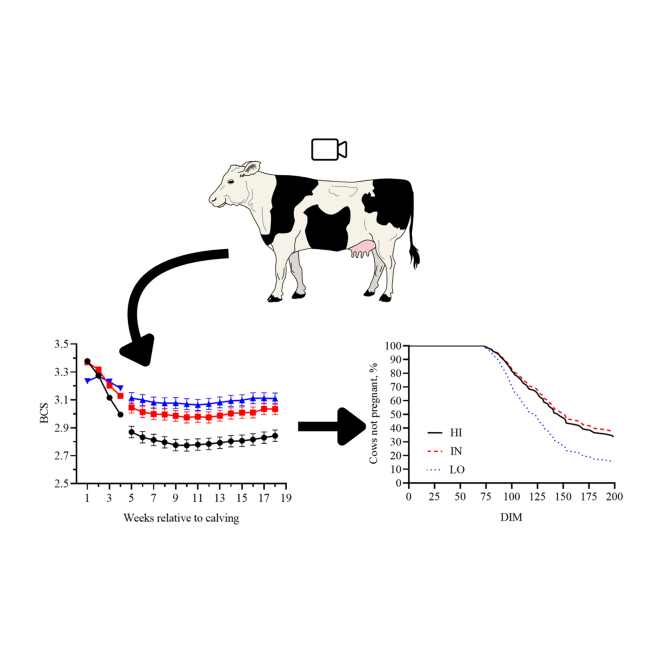
**Summary:** Body condition score (BCS) in early lactation reflects the negative energy balance and the mobilization of adipose tissue. Our objective was to evaluate the association between the automatically assessed postpartum BCS changes with milk yield, fertility, and health of Holstein dairy cows in a retrospective observational study. Cows were classified as having high (HI), intermediate (IN), and low (LO) BCS change in the first month of lactation. In this study, cows with greater milk yield experienced the greatest BCS loss and reduced fertility, whereas cows with minimal BCS loss had lower yield but improved reproductive performance. DIM = days in milk.

The transition period, which spans from 3 wk before to 3 wk after calving, is the most challenging phase of the entire lactation cycle in dairy cows (Drackley, 1999). The dramatic metabolic and hormonal changes occurring during this phase (Cattaneo et al., 2023) can affect health and productive and reproductive outcomes. During this time, due to the mismatch between energy requirements for production and energy intake from feed, cows often experience a loss in BCS, which reflects the mobilization of body fat reserves to support energy demands. Excessive BCS loss after calving has been associated with impaired metabolic status, reduced reproductive performance, and increased disease risk (Ferguson, 1996; Grummer, 2008). The severity of negative energy balance and the consequent reserve mobilization has a large interindividual variability (Carvalho et al., 2014), depending on several factors, such as, among the others, parity, genetic merit, health, milk yield. The magnitude of negative energy balance can affect reproduction. Of interest is that severe negative energy balance can impair the first ovulation and prolong the calving-to-conception interval (Diskin et al., 2003).

The evaluation of BCS is a useful technique to estimate the reserves of adipose tissue in dairy cows (Edmundson et al., 1989), allowing the monitoring of changes in body reserves and providing information on health and reproductive status of cows (Roche et al., 2009). Body condition score at calving and postcalving BCS loss have been reported to be related to reproductive success (Roche et al., 2009; Carvalho et al., 2014; Manríquez et al., 2021). Nevertheless, routine visual BCS assessment can be time consuming and is subjective to the evaluator's ability (Kristensen et al., 2006). In recent years, automated 3-dimensional (**3D**) camera technologies have been developed to quickly and objectively monitor BCS in dairy herds. These systems use images and depth sensors to capture the body shape as the animal walks past, providing repeatable, high-throughput BCS measurements without manual scoring bias. Such technology is increasingly available commercially and has been applied in research to investigate associations between BCS dynamics and reproductive outcomes (Pinedo et al., 2022). At the same time, blood progesterone is a valuable method for assessing the reproductive status of cows. It can be used to analyze ovarian activity, pregnancy status, and associations between progesterone levels during AI and pregnancy (Bruinjé et al., 2017). Laboratory techniques to measure blood progesterone are complex and labor-intensive, but milk progesterone concentration is closely associated with the concentration in blood (van de Wiel and Koops, 1986). New precision tools, such as cameras predicting BCS (Mullins et al., 2019) and automated in-line milk progesterone (**mP4**) analyzers (Bruinjé and Ambrose, 2019; Bruinjé et al., 2019), represent quick, practical, and noninvasive alternatives for monitoring the condition and reproductive status of dairy cows.

Previous studies have linked postpartum BCS dynamics with reproductive performance in dairy cows (Carvalho et al., 2014; Barletta et al., 2017), but most relied on visual BCS scoring and synchronization protocols, which may not capture the variability seen in herds with minimal use of synchronization. Automated phenotyping technologies provide opportunities to quickly and easily assess BCS and reproductive physiology. In particular, 3D camera systems can generate continuous objective estimates of BCS, whereas in-line milk progesterone monitoring enables high-resolution profiling of ovarian activity. Therefore, the objective of this study was to evaluate the association between changes in BCS, predicted by an automated 3D camera system, and postpartum reproductive outcomes derived from longitudinal milk progesterone profiles (i.e., commencement of luteal activity, resumption of ovarian cyclicity by the end of the voluntary waiting period [**VWP**], progesterone peaks) in a commercial Italian herd. Conventional reproductive performance metrics (i.e., pregnancy to first and second insemination, pregnancy up to 200 DIM, services per conception, time to conception) and production data were evaluated as secondary outcomes. We hypothesized that greater BCS loss in early lactation would be associated with delayed resumption of ovarian cyclicity and reduced reproductive performance. This study contributes to the growing body of research validating automated phenotyping technologies as innovative tools for herd management (Silva et al., 2021).

This retrospective observational study was carried out on a commercial farm in the province of Brescia (Italy). The farm milks on average 134 Holstein cows with an average milk yield of ∼37 kg/d, calving-to-conception interval of 135 d, and 21-d pregnancy rate of 22%. In the present study, cows calving over a 12-mo period (i.e., March 2022 to March 2023) were monitored. This was a convenience sample, as the study aimed to explore novel associations between postpartum BCS change measured by automated 3D camera technology, milk progesterone–derived reproductive outcomes, and conventional reproductive performance metrics in high-producing Holstein cows. The sample size was therefore determined by the number of eligible cows available in the herd during the study period, and no formal sample size calculation was performed.

Overall, data from 123 Holstein cows (70 were multiparous and 53 primiparous) were retrieved. Those cows were monitored from calving to 200 DIM. The farm is equipped with 2 automatic milking systems (VMS V310, DeLaval International AB, Tumba, Sweden), with sensors for evaluating mP4 levels (Herd Navigator; DeLaval International) and cameras for the automatic monitoring of BCS (DeLaval Body Condition Scoring system, DeLaval International). During the study period, cows visited the AMS 2.5 times per day, on average. The mP4 analysis system is based on a bio-model that automatically collects milk samples, measures mP4 using a dry-stick enzyme immunoassay technique, and stores data on both raw (actual) and adjusted (smoothed) mP4 levels, at algorithm-driven intervals after calving (Bruinjé and Ambrose, 2019). The system begins monitoring mP4 levels at 20 DIM (Bruinjé et al., 2019). If mP4 remains low until 45 DIM, an alert for prolonged anestrus is triggered. The system continues sampling until the first ovulation, at which point it recognizes the cow as cyclic and calculates the date of the next estrus. After the first estrus alert, samples are taken on d 5, 9, 14, and 18 of the cycle. From d 18 onward, samples are collected daily until the mP4 drops below 5 ng/mL, signaling a new estrus alert and starting a new cycle. On average, 10 to 15 samples are collected per cycle.

The BCS monitoring system consists of a 3D camera positioned above the exit gate of the milking robots, previously validated by Mullins et al. (2019). The BCS of all lactating cows is measured daily after the first milking and before feeding. The score is reported in increments of 0.1 on a scale from 1 (emaciated) to 5 (obese). In this study, data from calving to 120 DIM were retrieved. Data were averaged by week. For multiparous cows only, BCS data from the last 30 d of the previous lactation were collected. The postpartum BCS nadir and the time to nadir were calculated for each cow.

Once daily, cows were fed a TMR based on wheat silage, high moisture corn, red clover silage, protein concentrate, alfalfa haylage, soybean meal, and straw. Concentrate was offered by the milking robot, with the allowance increasing from calving (2 kg/d) to the maximum allowance at 30 DIM (4.5 kg). Data about milkings were retrieved from the automatic milking system software up to 120 DIM and averaged by week.

Starting from 3 DIM, all cows are inspected and tested for blood BHB with strips biweekly (Nova Vet, Nova Biomedical Italia S.r.l., Italy). When BHB was greater than 0.8 mmol/L, 350 mL of propylene glycol were administered orally once daily for 3 d. Health status was checked by the farm personnel and veterinarian, and diseases and disorders (i.e., ketosis, metritis, mastitis, and lameness) and their diagnosis dates were recorded in the farm management software. Ketosis was defined as blood BHB >1.4 mmol/L, metritis diagnosed when mucopurulent and foul-smelling discharge was detected, mastitis diagnosed upon inspection following an automatic milking system alarm (triggered by changes in conductivity, milking intervals, and blood content), and lameness was diagnosed by visual observation. Morbidity was evaluated in the first 120 DIM and defined as cows having at least one of the aforementioned diseases and disorders. Between 10 and 20 DIM and at 70 DIM, cows were checked by the farm veterinarian to assess uterine and reproductive status by rectal palpation. After a VWP of 75 d, estrus detection was based on mP4 alarms. Cows showing reproductive abnormalities at the 70 DIM check (17% of the monitored cows), including delayed resumption of cyclicity or uterine disorders, were enrolled in a Double-Ovsynch protocol (Souza et al., 2008). Artificial insemination (**AI**) was performed on average 53 ± 8 h (SD) after the estrus alarm (mP4 < 5 ng/mL). Pregnancy checks were performed at 35 and 70 d after AI if mP4 did not decline before. Pregnancy was diagnosed at 35 ± 5 d after AI and confirmed at 70 ± 5 d after AI by per uterine ultrasonography and calculated as the number of cows diagnosed pregnant divided by the total number of cows that received AI. Only data from pregnancy confirmation (70 ± 5 d after AI) was used for further analysis. Cows were followed up until 200 DIM, and time to pregnancy was defined as DIM at the AI that resulted in a pregnancy. We evaluated the odds of a pregnancy, the proportion of pregnant cows, and the DIM at the first and second AI, as well as the cumulative odds of pregnancy and the proportion of pregnant cows to first 2 AI. The time to pregnancy and the cumulative pregnancy up to 200 DIM were then also evaluated.

Profiles of mP4 were evaluated between the first postpartum sample (20.1 ± 0.5 DIM) and the first AI (96.2 ± 17.8 DIM). The commencement of luteal activity (**CLAC**) was defined as the DIM of the first of at least 2 consecutive samples with mP4 ≥5 ng/mL after calving (Bruinjé et al., 2017). Cows having CLAC by the end of the VWP (i.e., 75 DIM) were defined as having resumed ovarian cyclicity. For cows not having it by 120 DIM (n = 1/group), CLAC was not calculated. Based on the visual inspection of mP4 profiles, the mP4 peak was defined as the highest mP4 recorded in the last 8 d of the luteal phase preceding an AI event, and mP4 pre-AI was defined as the single record of mP4 <5.0 ng/mL preceding AI (Bruinjé et al., 2019). Overall, 12 cows were excluded from this analysis because of missing mP4 data.

Regardless of parity, cows were retrospectively classified into tertiles based on BCS change (**ΔBCS**) from calving to 30 DIM, as an indicator of body reserve mobilization in the first month of lactation. The endpoint of 30 DIM was selected as an arbitrary cut-off. The average BCS value of 3 consecutive days was used (i.e., d 0, 1, and 2 for the calving measure and d 29, 30, and 31 for the 30 DIM measure). The first ΔBCS tertile (low ΔBCS [**LO**]) included 18 primiparous and 23 multiparous cows, the second tertile (intermediate ΔBCS [**IN**]) included 17 primiparous and 24 multiparous cows, and the third tertile (high ΔBCS [**HI**]) included 18 primiparous and 23 multiparous cows. During the study period, 2 cows (1 in HI and 1 in IN) were removed from the herd. Their data were included in the analysis up to the date of removal.

Weekly averages of BCS, milk yield, and milking frequency from 30 to 120 DIM were analyzed with repeated measures mixed models, including the fixed effects of ΔBCS tertile, weeks in milk (**WIM**), and their interaction, parity (i.e., primiparous [parity = 1] vs. multiparous [parity ≥2]), and the random effect of the cow. Models for milk yield and milking frequency included also the fixed effects of the AMS unit. Model assumptions (normality and homogeneity of residuals) were checked using residual plots. Data with a single measurement (i.e., BCS nadir, time to BCS nadir, CLAC, mP4 peaks, and mP4 before AI) were analyzed with ANOVA including the fixed effects of tertile and parity. Results were reported as LSM and 95% CI. Binary outcomes (i.e., resumption of ovarian cyclicity at the end of the VWP, pregnancy at first and second AI, cumulative pregnancy at first and second AI, and pregnancy within 200 DIM) were analyzed by logistic regression using the GLIMMIX procedure of SAS (version 9.4, SAS Institute Inc., Cary, NC), including the effects of BCS tertile, parity, and synchronization (yes/no). Results were reported as adjusted probabilities, odds ratios (**OR**), and their 95% CI. Time-to-event outcomes were analyzed via Cox's proportional hazard model using the PHREG procedure of SAS and Kaplan–Meier survival curves using the LIFETEST procedure of SAS. The proportional hazards assumption was verified using the ASSESS statement with plots of martingale and Schoenfeld residuals. No violations of the proportional hazards assumption were observed. For analyses of time to pregnancy, cows were right-censored when they left the herd before 200 DIM or were nonpregnant at 200 DIM. Results are reported as the adjusted hazard ratio (**AHR**) and 95% CI. The *P*-values for all pairwise comparisons were adjusted using the Tukey–Kramer method. Differences with *P* ≤ 0.05 were considered significant, and when *P* ≤ 0.10, differences were discussed in the context of tendencies.

By design, ΔBCS in the first 30 DIM was the greatest in HI and the lowest in LO (−0.47 ± 0.09 vs. −0.30 ± 0.03 vs. −0.09 ± 0.15, mean ± SD), with primiparous cows tending to lose more BCS than multiparous (–0.30 ± 0.13 vs. −0.27 ± 0.22). Overall, BCS between 30 and 120 DIM mirrored ΔBCS, with increasing body condition from HI to LO (2.80 [2.73–2.89] vs. 3.00 [2.92–3.08] vs. 3.09 [3.01–3.17]; *P* < 0.01), with a different course over time (ΔBCS × WIM, *P* < 0.01), as shown in Figure 1. In multiparous cows, the BCS during the last 28 d before dry-off was not different among groups (3.39 [3.24–3.54] vs. 3.39 [3.26–3.52] vs. 3.32 [3.19–3.45] in HI, IN, and LO, respectively; *P* = 0.69). The nadir in postpartum BCS was lower in HI compared with IN and LO (2.74 [2.66–2.83] vs. 2.93 [2.85–3.01] vs. 2.99 [2.91–3.07]; *P* < 0.01) but no significant difference was noted in the time taken to reach it (64.8 [56.2–73.4] vs. 62.8 [54.2–71.5] vs. 55.2 [46.6–63.8] DIM; *P* = 0.25), despite the numerically lower value in LO. Overall, milk yield followed the ΔBCS, being lower in LO compared with IN and HI (44.8 [42.6–47.0] vs. 43.7 [41.6–45.8] vs. 40.7 [38.6–42.8] kg/d; *P* = 0.02), whereas the ΔBCS × WIM interaction was not significant (*P* = 0.44). Milking frequency was not different among groups (2.64 [2.44–2.85] vs. 2.71 [2.51–2.91] vs. 2.62 [2.42–2.82] milkings/d; *P* = 0.78).

**Figure 1:**
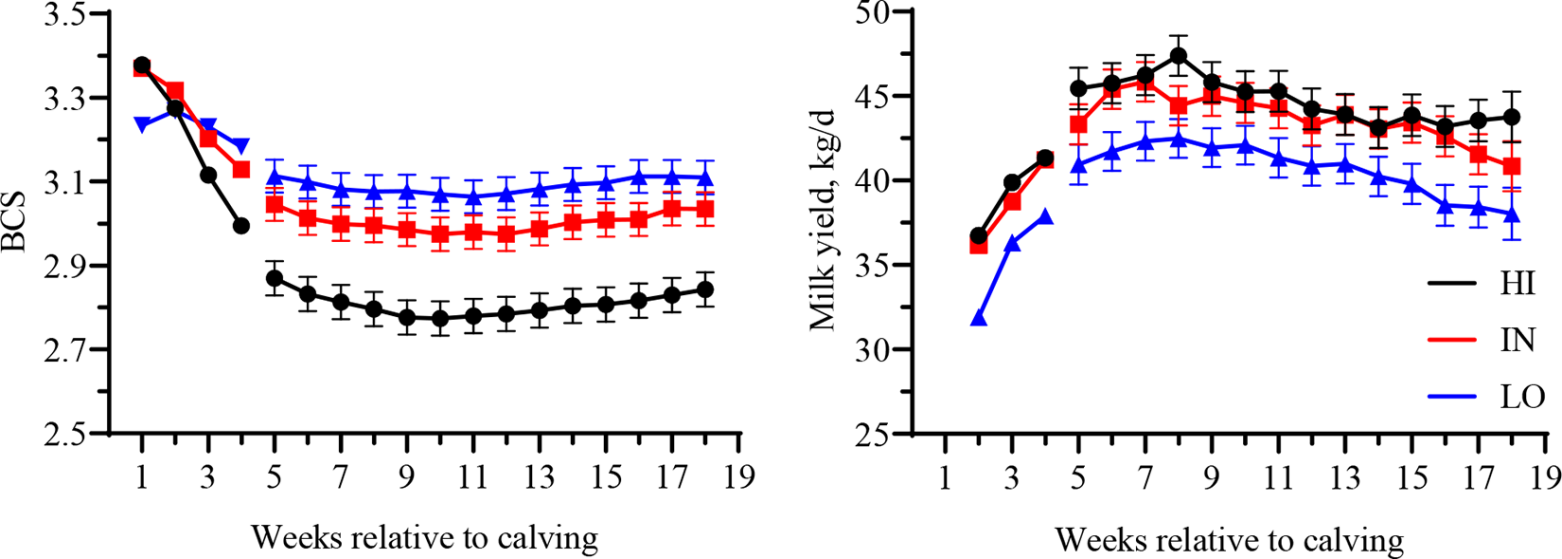
Weekly averages of postpartum BCS and milk yield in cows with high (HI), intermediate (IN), or low (LO) BCS change (ΔBCS; n = 41/group) from 0 to 30 DIM in one Italian dairy farm. Error bars are SE.

The CLAC tended to differ among tertiles (*P* = 0.10; Table 1), with HI having later CLAC than other groups, but no differences were noted in terms of the percentage of cows resuming ovarian cyclicity before the end of the VWP. Similarly, mP4 peaks and the subsequent declines before the first 2 AI did not highlight any differences. The proportion of cows pregnant at first AI did not differ, but LO cows tended to be more likely to become pregnant at the second AI compared with IN (OR = 3.19, 95% CI = 0.91–11.24; *P* = 0.12). Thus, the cumulative proportion of pregnant cows at the first and second AI tended to be greater in LO compared with HI (OR = 2.83, 95% CI = 1.09–7.34) and IN (OR = 2.52, 95% CI = 0.99–6.37; *P* = 0.06), and the proportion of pregnant cows up to 200 DIM was greater (*P* = 0.04). Compared with LO, HI (AHR = 0.56, 95% CI = 0.33–0.93) and IN (AHR = 0.52, 95% CI = 0.30–0.87) cows had reduced hazard of pregnancy up to 200 DIM (Figure 2). Morbidity was not significantly different among tertiles (*P* = 0.15).

**Table 1:** Least squares means of features derived from milk progesterone profiles and proportions of pregnancy outcomes in Holstein cows with high (HI), intermediate (IN), or low (LO) BCS change (ΔBCS; n = 41/group) from 0 to 30 DIM in one Italian dairy farm

Item	ΔBCS^1^			SEM	*P*-value
	HI	IN	LO		
CLAC,^2^ d	44.2 (36.5–51.8)	36.12 (28.3–43.9)	35.9 (28.0–43.8)	3.98	0.10
Resumption ovarian cyclicity by VWP, %	90.0 (33/38)	97.8 (33/35)	98.7 (37/38)	4.94	0.22
Milk progesterone peak at first AI,^3^ ng/mL	21.7 (19.1–24.3)	21.2 (18.7–23.6)	22.9 (20.4–25.4)	1.05	0.44
Milk progesterone nadir before first AI,^3^ ng/mL	3.44 (3.11–3.77)	3.48 (3.17–3.79)	3.77 (3.45–4.08)	0.16	0.15
Milk progesterone peak at second AI,^3^ ng/mL	21.2 (17.7–24.7)	19.1 (15.7–22.6)	20.1 (16.8–23.4)	1.73	0.56
Milk progesterone nadir before second AI,^3^ ng/mL	3.36 (2.95–3.78)	3.57 (3.15–4.00)	3.71 (3.32–4.11)	0.21	0.32
Pregnancy at first AI, %	23.8 (9/40)	31.3 (12/41)	36.3 (14/41)	9.20	0.52
Pregnancy at second AI, %	30.5 (8/29)	25.4 (6/27)	52.1 (13/27)	11.30	0.12
Cumulative pregnancy at first and second AI, %	46.9 (17/41)	49.8 (18/41)	71.4 (27/41)	8.84	0.06
Cumulative pregnancy up to 200 DIM, %	68.7 (28/41)	59.1 (24/41)	85.6 (35/41)	7.91	0.04
Services per conception, n	2.11 (1.58–2.80)	1.78 (1.28–2.46)	1.68 (1.24–2.29)	0.30	0.44
Morbidity,^4^ %	24.92 (11/41)	15.24 (7/41)	8.69 (4/41)	7.04	0.15

1 The HI, IN, and LO BCS groups were defined by tertiles of ΔBCS from 0 to 30 DIM. The HI group included cows with the highest ΔBCS values (range: −0.78 to −0.36), the IN group included intermediate ΔBCS values (−0.35 to −0.25), and the LO group included the lowest ΔBCS values (0.24–0.41). Continuous data are reported as LSM and 95% CI (in parentheses), and binary data as adjusted probabilities and proportions.

2 Before CLAC, 3 cows in the HI group underwent a Double-Ovsynch protocol.

3 Milk progesterone peak is the highest value recorded in the last 8 d of the luteal phase preceding an AI event, and milk progesterone nadir before AI is the single record of mP4 <5.0 ng/mL preceding AI.

4 Ketosis: 2/41 in HI, 0/41 in IN, 1 /41 in LO; mastitis: 2/41 in HI, 1/41 in IN, 1/41 in LO; metritis: 0/41 in HI, 3/41 in IN, 0/41 in LO; lameness: 7/41 in HI, 3/41 in IN, 2/41 in LO. Morbidity in first 30 DIM was 7/41 in HI, 6/41 in IN, and 1/41 in LO.

**Figure 2:**
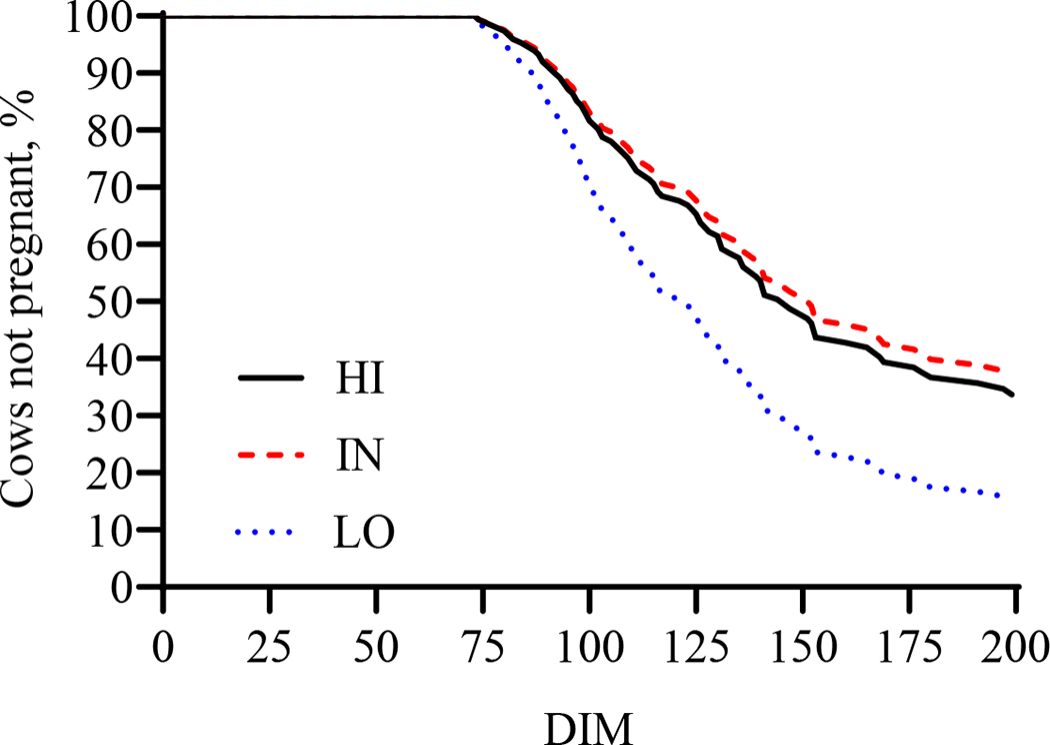
Kaplan–Meier survival curves for time to pregnancy by 200 DIM in cows with high (HI), intermediate (IN), or low (LO) BCS change (ΔBCS; n = 41/group) from 0 to 30 DIM in one Italian dairy farm. Median and mean days to pregnancy: HI = 147 and 151; IN = 141 and 145; LO = 121 and 130. Percentage of cows censored: HI = 31.7; IN = 41.4; LO = 14.6. Effect of ΔBCS group: *P* = 0.04.

In the present study, we stratified cows into tertiles based on the BCS change in the first month of lactation, as predicted by an automated 3D camera system. Cows with low BCS loss (LO) were already thinner at calving than cows losing more BCS (HI and IN). Several studies previously reported this association (Dechow et al., 2002; Carvalho et al., 2014; Barletta et al., 2017). In our study, the BCS of LO cows at calving was within the optimal range (3.00–3.25) reported by Roche et al. (2009), whereas IN and HI had slightly higher values than the upper limit proposed. Classifying cows based on ΔBCS in the first 21 DIM, Barletta et al. (2017) reported nondifferent yields in cows gaining or losing BCS. Results from our study suggest that the greater yield observed in HI was associated with a greater mobilization from body reserves and that those cows were likely in a greater negative energy balance than LO (Roche et al., 2006). The observational design of our study prevents understanding whether greater productivity, possibly driven by genetic merit or other factors not investigated here, contributed to the increased BCS loss in HI. Unfortunately, genetic information for the cows included in this study was not available. The unexpected slightly greater ΔBCS in primiparous cows compared with multiparous cows was likely due to their higher BCS at calving (3.45 vs. 3.25), which made them more prone to mobilizing body reserves and consequently losing BCS. Among the other factors possibly related to BCS loss, morbidity, although not significantly, was greater in HI and LO, particularly in the first 30 DIM. This suggests a possible bidirectional relationship, where diseases may have contributed to BCS loss, or conversely, BCS loss may have predisposed cows to diseases.

The reproductive outcomes of this study agree with previous works (Barletta et al., 2017; Manríquez et al., 2021) reporting earlier first ovulation and improved reproductive success in cows gaining BCS or minimally losing BCS postpartum compared with cows experiencing severe BCS loss. Most previous studies evaluated the association between ΔBCS and reproductive performance using synchronized estrus protocols (Carvalho et al., 2014; Barletta et al., 2017). In contrast, our study assessed reproductive outcomes primarily through natural estrus, offering a different perspective. Moreover, by deriving reproductive outcomes directly from longitudinal in-line milk progesterone profiles, we captured ovarian dynamics with higher resolution than traditional estrus detection or farm records. In our study, cows in the HI group tended to have later CLAC compared with IN and LO cows, and no differences were observed in the proportion of cows resuming ovarian cyclicity before the end of the VWP. Cows without a CLAC before 120 DIM were excluded from the analysis (n = 1/group), which may introduce survival bias, as cows with the most extreme delays in cyclicity were not included. This could lead to underestimation of the association between BCS loss and delayed CLAC. Although the use of synchronization protocols was included in the models, the use of hormonal treatments, despite being limited, may still have influenced the timing of CLAC, potentially reducing differences attributable solely to spontaneous ovarian activity. Despite these limitations, the observed trend supports the hypothesis that greater BCS loss is associated with delayed resumption of ovarian activity, consistent with the patterns of reproductive performance noted in this herd. Similarly, in a larger study (Bruinjé et al., 2023), a BCS loss from prepartum to 9 WIM greater than 0.5 points was associated with reduced spontaneous estrus detection. No significant associations were observed between BCS change and the other milk progesterone variables. Severe negative energy balance has been suggested to impair luteal function. In our dataset, cows with greater BCS loss tended to have delayed CLAC, but progesterone peaks and nadirs at estrus were not significantly affected. These findings suggest that although severe BCS loss may be associated with the timing of ovarian activity resumption, it was not measurably related to the progesterone dynamics investigated under the conditions of this commercial herd. Future studies with larger sample sizes or specifically designed to investigate this association may be required to fully detect subtle effects of energy balance on progesterone variables.

Cows with the greatest BCS loss had the highest milk yield, consistent with the findings of Manríquez et al. (2021), but indications of reduced reproductive performance, particularly at first AI. Conversely, cows gaining (or losing minimal) BCS had the greatest pregnancy rate but the lowest milk yield. Strategies are therefore warranted to improve the energy balance in the postpartum period while supporting milk synthesis and reproduction and enabling cows to express their genetic potential.

The outcomes of this observational retrospective study should be interpreted with caution due to several limitations. The study was conducted on a single farm, included a limited number of animals, and covered only one production year. This small and context-specific dataset may reduce the robustness of the associations identified, as the limited sample size increases the likelihood of random variability influencing the results. Moreover, the findings may not be broadly generalizable to other herds that differ in management, environment, or genetic background, thereby limiting their external applicability. Nonetheless, this study demonstrates the feasibility and value of using automated phenotyping technologies, including 3D camera–derived BCS and in-line milk progesterone monitoring, in commercial dairy systems. The integration of these systems allowed us to investigate continuous, hormone-derived measures of ovarian function that are less frequently explored than binary reproductive outcomes. Our findings demonstrate the potential of these technologies to capture biologically relevant associations and to provide early insights into postpartum physiology.

This work provides exploratory evidence on the trade-offs between energy balance, milk yield, and reproduction, measured by automated phenotyping technologies in commercial conditions. Future studies with larger and more diverse herds are warranted to strengthen these associations and to further explore whether severe postpartum BCS loss is physiological or driven by underlying metabolic or management-related factors.
